# Foreign body granuloma development after calcium hydroxylapatite injection for stress urinary incontinence: A literature review and case report

**DOI:** 10.1080/2090598X.2022.2146859

**Published:** 2022-11-15

**Authors:** David A. Csuka, John Ha, Andrew S. Hanna, Jisoo Kim, William Phan, Ahmed S. Ahmed, Gamal M. Ghoniem

**Affiliations:** a Department of Urology, University of California Irvine, CA, United States; b Department of Computational & Systems Biology, University of California Los Angeles, CA, United States; c Department of Psychological and Brain Sciences, University of California Santa Barbara, CA, United States; d Department of Urology, Aswan University, Aswan Egypt

**Keywords:** Urinary stress incontinence, lower urinary tract symptoms, bulking agent, calcium hydroxylapatite

## Abstract

**Objectives:**

To present a case of foreign body granuloma (FBG) development after injection of calcium hydroxylapatite as a urethral bulking agent and to review all documented cases of this phenomenon in the literature.

**Methods:**

We analyzed a new case of calcium hydroxylapatite-induced FBG. We also conducted a literature review of the PubMed, Embase, CINAHL, and Web of Science databases through March 2022. Reports were included if they contained stress urinary incontinence patients that developed an FBG after calcium hydroxylapatite injection. The cases were reviewed for presenting symptoms, patient demographics, granuloma details, and surgical treatment.

**Results:**

We screened 250 articles and included six articles between 2006 and 2015 in addition to the present case. The median age of the patients was 65.5 years (range 45–93), and all patients were female. The most common presenting symptoms and the proportion of patients affected were difficulty voiding (4/8), recurrent urinary incontinence (3/8), and dyspareunia (2/8). The median time between the first CaHA injection and discovery of the FBG was 5 months (range 1–50). The median longest dimension of the FBGs was 1.85 cm (range 1.0–3.0). The 8 masses observed were evenly distributed throughout the urethra, with 3 in the bladder neck, 2 in the midurethra, and 3 in the distal urethra. Surgical excision was the predominant management choice, with some variation in technique.

**Conclusions:**

Severe, persistent lower urinary tract symptoms after calcium hydroxylapatite injection may indicate an FBG, which has been successfully managed with surgical excision.

## Brief Summary

A case presentation of foreign body granuloma development after calcium hydroxylapatite injection alongside a review of all such instances in the literature.

## Introduction

Stress urinary incontinence (SUI) is the involuntary loss of urine upon increased abdominal pressure or physical movement, and it often occurs secondary to intrinsic sphincter deficiency (ISD) [1]. The annual incidence of SUI is estimated to be between 4% and 10% [2]. First-line treatments for SUI involve primarily behavioral modifications, and possible surgical interventions include the midurethral sling (MUS) and the urethral bulking agent (UBA). UBAs are recommended for women who seek a less invasive SUI treatment with lower risk and shorter recovery time compared to a MUS, if they understand that UBAs often require repeat injections [3]. UBAs are typically injected into multiple sides of the midurethra or proximal urethra, and they treat SUI by stimulating endogenous collagen production, thus increasing urethral coaptation and resistance. Four UBAs are currently approved in the United States, including calcium hydroxylapatite, polydimethylsiloxane, polyacrylamide hydrogel, and carbon-coated zirconium oxide [4]. Approved by the FDA in 2005, calcium hydroxylapatite (CaHA, Coaptite™) is a non-pyrogenic UBA composed of spherical particles of CaHA (75–125 micron diameter), a sodium carboxymethylcellulose carrier gel, glycerin, and sterile water [5].

Common UBA complications and adverse events include urinary tract infection, urinary retention, urgency, urge incontinence, pain, dysuria, hematuria, infection, cystitis, and other lower urinary tract symptoms (LUTS). Complications that occur more rarely are urethral foreign body granuloma (FBG), urethral prolapse, fistula, UBA migration, urethral erosion, and vaginal wall erosion [6]. Foreign body reactions occur to some extent at the interface between native tissue and all artificial implants and prostheses, but high severity in both the immune cell activity as well as the acute and chronic stages of inflammation can lead to the diagnosis of an FBG. FBGs are histologically characterized by the presence of Langhans or foreign body type multinucleated giant cells derived from the fusion of macrophages with each other at the interface of the native tissue and material [7,8]. FBGs caused by UBAs have not been known to involve material extrusion, but they can coincide with UBA calcification.

We encountered a new case of an obstructive urethral FBG upon CaHA UBA injection. As this complication is rare, there are inadequate diagnostic and treatment-related guidelines available. Previous reviews featuring CaHA-related FBG cases have all been part of general UBA reviews and are either lacking in detail or incomprehensive [4,6,9]. Our objectives were to report a novel case and to conduct a literature review of CaHA-related FBGs.

## Case Report

A 45-year-old female presented to the urogynecology clinic and was diagnosed with SUI, dyspareunia, stage II uterovaginal anterior wall prolapse (cystocele), and exposure of the implanted MUS mesh located 4 cm proximal to the introitus just right of the midline. Afterwards, the patient had a total vaginal hysterectomy, bilateral salpingectomy, uterosacral ligament suspension, anterior and posterior repair, MUS excision, and cystoscopy with injection of calcium hydroxylapatite (CaHA, Coaptite^TM^, Boston Scientific: 890–300), a particulate UBA.

Intraoperative cystourethroscopy showed cystitis cystica and was negative for immediate bulking agent extrusion or erosion. Three days after the procedure, she reported incomplete emptying and persistent lower abdominal pain, which she described as a constant burning sensation that did not change with activity. She developed an increased post-void residual (PVR) urine volume of 150 mL and a urinary tract infection, which was treated. She continued with a urinary frequency of every 40 to 60 minutes. One month after the procedure, she had painful intercourse. She also reported a painful bladder when full that disappeared after urination. The patient described occasional urinary leakage with coughing and sneezing, which had improved since the UBA administration.

**Figure 1.**
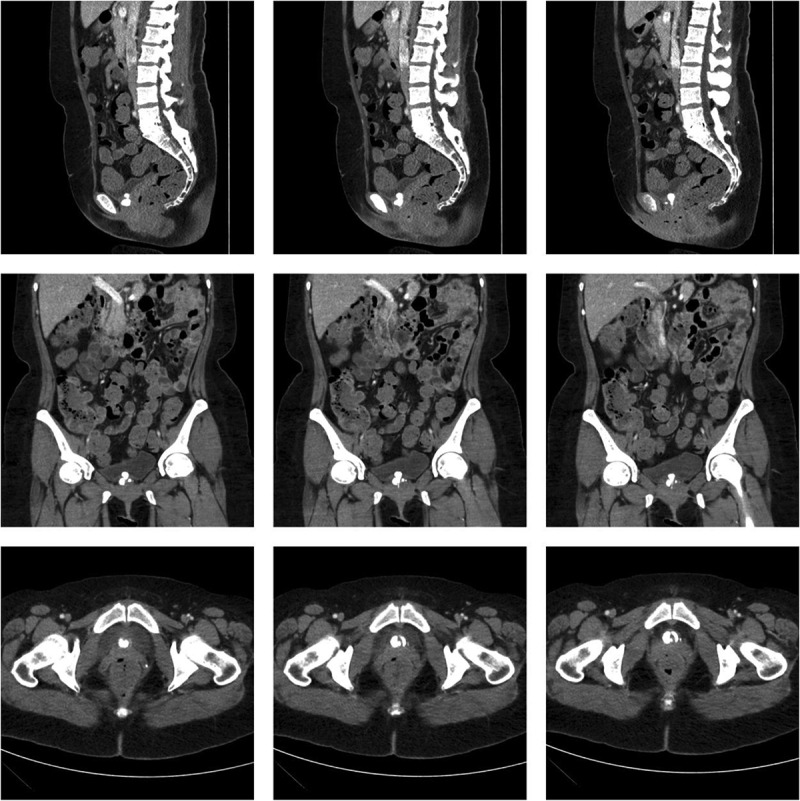
Sagittal, coronal, and axial CT scans of the abdomen and pelvis showing the foreign body granuloma, which can be misinterpreted as a bladder calcification or stone.

**Figure 2.**
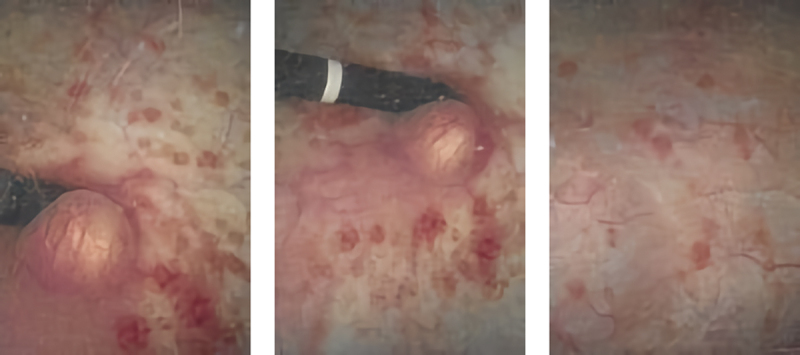
Flexible cystourethroscopy imaging of the patient’s Coaptite-induced bladder neck mass before and after the transurethral resection.

She was then referred to our female urology division for a second opinion and possible bladder biopsy. Her genitourinary history consisted of urgency, occasional SUI and urge urinary incontinence (UUI), difficulty emptying the bladder, dysuria, dyspareunia, nocturia three to four times per night, and daytime urinary frequency of every 90 minutes. A CT scan of the abdomen and pelvis performed 50 days after the bulking agent injection showed two potential calcifications or masses (1.9x1.1 and 1.0 × 1.4 cm) at the bladder neck and posterior urethra (Figure 1), which are often indicative of bladder stone formation. However, flexible cystourethroscopy showed cystitis cystica and a ball-shaped bladder neck mass arising from the proximal urethra (Figure 2), leading us to suspect a CaHA-induced foreign body granuloma. The patient underwent urodynamic studies and was found to have bladder outlet obstruction (BOO) with mild PVR. Her detrusor pressure was 45 cmH2O with a maximum flow rate of 5 mL/sec. We then performed transurethral resection (TUR) and foreign body removal. A resectoscope was introduced into the patient’s urethra, and the mucosa over the granuloma was initially resected with a bipolar loop exposing the calcified CaHA particles. With gentle pressure on the granuloma, the particles were slowly expressed into the bladder then evacuated. A superficial resection was performed to remove the remaining particles stuck to the granuloma base, and special care was taken to avoid damaging the bladder neck muscles, which can serve as a continence mechanism. An indwelling Foley catheter was left in place for one week. The patient recovered well with greatly improved urination, no residual urine, less pain, and mild SUI. Three months later, flexible cystourethroscopy showed complete healing of the bladder neck and no residual particles.

## Materials and Methods

**Figure 3.**
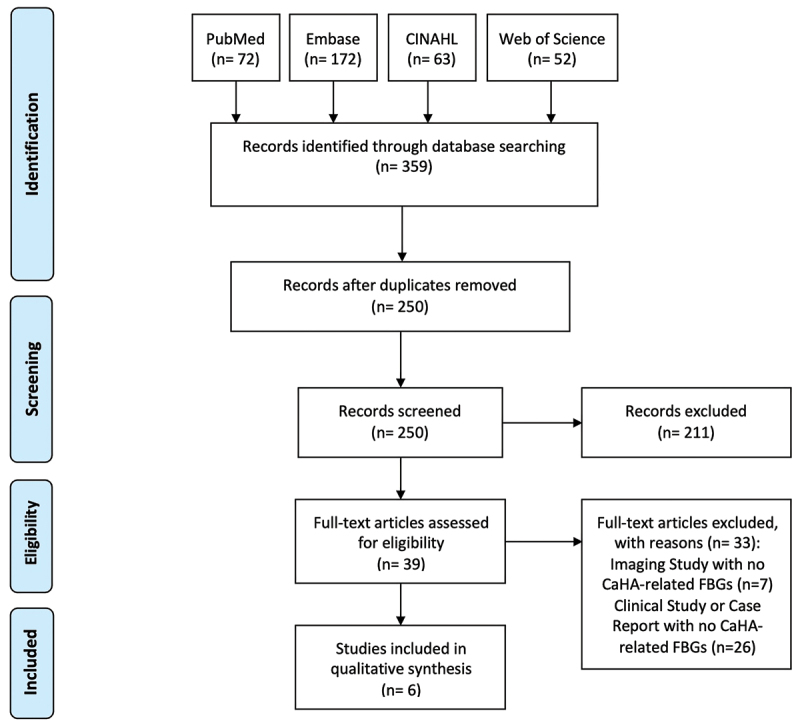
Flow diagram of the literature review process.

This literature review of FBGs and urethral masses upon injection of CaHA involved searching six databases, with a flow diagram for reproducibility (Figure 3). A literature search of the PubMed, Embase, CINAHL, and Web of Science databases was conducted on 1 March 2022. The search terms utilized for each database are as follows: PubMed: (coaptite OR ‘calcium hydroxylapatite’ OR ‘calcium hydroxyapatite’) AND (urin* OR urogenital diseases OR urogenital system); Embase: (coaptite OR ‘calcium hydroxylapatite’ OR ‘calcium hydroxyapatite’) AND (urin* OR ‘urogenital tract disease’/exp); CINAHL: (coaptite OR ‘calcium hydroxylapatite’ OR ‘calcium hydroxyapatite’) AND (urin* OR (MH ‘Urologic Diseases+’)); Web of Science: (coaptite OR ‘calcium hydroxylapatite’ OR ‘calcium hydroxyapatite’) AND (urin*). MeSH and Emtree query enhancements were utilized to broaden the range of articles evaluated. Additionally, ClinicalTrials.gov and the Manufacturer and User Facility Device Experience (MAUDE) adverse event database were queried for relevant CaHA reports. Articles were reviewed and assessed for inclusion independently by two authors (DC and JH). Eligible articles documented urethral FBGs or masses that both resulted from CaHA UBA injection and required surgical intervention, although to be exhaustive we discuss all asymptomatic cases encountered that may potentially refer to mild FBGs as they are informative and difficult to find. Cases of CaHA used as a vesicoureteral reflux treatment, fecal incontinence treatment, or dermal filler were excluded. Authors were contacted via correspondence for important missing information. Patient history, injection details, surgical outcome, and treatment plan were obtained from the included reports.

The following information was abstracted from the eligible articles when available: year, first author, patient age, time between first injection and discovery of mass, granuloma location, granuloma size, injection amount, time between injections, and injection position. The main objective was to comprehensively characterize all known CaHA-related FBGs, thus allowing surgeons, urogynecologists, and providers using Coaptite to better understand the associated risks andappropriate treatment courses for FBGs of various morphologies.

## Literature Review Results

Two hundred and fifty deduplicated articles were screened, 39 were assessed for eligibility, and six were included in this review (Figure 3). Nonrelevant studies or those which included CaHA UBA treatments administered to patients with no reports of urethral FBGs or masses were formally excluded but mentioned if potentially instructional for urogynecologists.

All six articles were case reports, and they included seven total patients, meaning that eight CaHA-related FBG cases are known including ours (Table 1). Not a single instance of a Clavien Grade III FBG was reported in the 1449 total CaHA patients from the 33 unincluded articles assessed for eligibility and a currently unpublished post-approval clinical trial [10], which sheds light on the rarity of this complication. The closest presentation available which did not require surgical intervention was described by Heredia et al. as an ‘asymptomatic distal urethral polyp’ [11]. Additionally, Goltzman et al. report an unspecified case of either CaHA vaginal erosion or a local inflammatory response that required transvaginal excision, potentially indicating an FBG [12]. Outside of common CaHA complications, including urinary tract infection, transient urinary retention, urge incontinence, and urgency, Mayer et al. reported two cases of treatment-related adverse events in their clinical trial: CaHA migration to the distal urethra causing vaginal wall erosion and CaHA migration into the bladder trigone [13].

**Table 1.** All 8 cases of CaHA-induced FBGs in the medical literature including ours. *Estimated to be over 12 months later by Elmhishi et al. per correspondence.

Year	First Author	Patient Age	Time Between First Injection and Discovery of Mass (months)	Presenting Symptoms	Granuloma Location	Granuloma Size (cm)	Injection Amount (mL) and Time Between Multiple Injections	Injection Position (o’clock)
2006	Palma^14^	55	3	Urinary incontinence	Distal urethra	3.0	2.5	3, 6, 9
2007	Ko^15^	74	1	Urinary incontinence, hematuria	Distal urethra	N/A, 2 granulomas	2.0	2, 10
2008	Lai^16^	67	7	Difficulty voiding	Distal urethra	2.0	2.0	3, 9
2011	Gafni-Kane^17^	65	2	Dyspareunia	Bladder neck	(1.4x1.8x1.0), (1x1)	(2.0)-1 month-(1.0)-1 month-(1.0)	(5, 7), (4, 6), (7)
2011	Gafni-Kane^17^	53	20	Urinary incontinence	Midurethra	1x2.5x1	(2.0)-1 month-(1.0)-1 month-(1.0)	(5, 7), (5), (2, 5)
2014	Schrop^18^	93	50	Difficulty voiding/urinary retention	Midurethra	1.0x1.0	(2.0)-5 months-(3.0)	N/A
2015	Elmhishi^19^	66	N/A*	Difficulty voiding, fatigue, nausea, vomiting, hypotension, hypovolemia	Bladder neck	1.1	N/A	N/A
2022	Ghoniem	45	1.6	Difficulty voiding, dyspareunia, cystitis cystica	Bladder neck	1.9x1.1	2.0	N/A

The median age of the patients was 65.5 years (range 45–93), and all patients were female. The median time between the first CaHA injection and FBG discovery was 5 months (range 1–50). The median longest dimension of the FBGs was 1.85 cm (range 1.0–3.0). The 8 masses observed were evenly distributed throughout the urethra, with 3 in the bladder neck, 2 in the midurethra, and 3 in the distal urethra. The most common presenting symptoms and the fraction of patients affected were difficulty voiding (4/8), recurrent urinary incontinence (3/8), and dyspareunia (2/8). (Table 1) The FBGs were all excised, either with a circumferential incision around the base or longitudinal incision with bead explantation prior to resection and urethral suturing.

Palma et al. describe a case of SUI developing after surgery for a pelvic bone fracture. CaHA was injected and a large 3.0 cm distal urethral mass formed, prolapsing through the urethral meatus during urination. The FBG was removed with an incision around its base, and the patient became continent with a fascia sling [14]. Ko et al. present of case of SUI development after a distal urethrectomy for urethral squamous cell carcinoma. In addition to two distal urethral masses and urethral prolapse, the chief postoperative symptoms were recurrent incontinence and gross hematuria. The two FBGs were found just one month after the procedure and were also circumferentially resected in a transurethral fashion [15]. The patient in Lai et al. remained with SUI after two failed slings, and a distal urethral CaHA-related FBG developed, soon prolapsing through the meatus. Instead of a circumferential resection, the mass was incised, the CaHA particles were removed, and the edges of the urethral mucosa were marsupialized to the vaginal mucosa much like a Spence diverticulum procedure. The SUI persisted after a successful surgery, and the patient became continent after three more urethral CaHA injections [16]. Given that susceptibility to CaHA FBGs likely has a patient-specific immune component, we believe that continuing to administer the same UBA at any time after development of an FBG is an unwise decision despite this positive outcome. In the first case of the Gafni-Kane et al. article, a second suburethral mass appeared one month after the excision of the first. The second mass was incised to remove the CaHA then resected. The patient became continent after receiving two CaHA injections 9 and 10 months postoperatively. In the second case, the CaHA FBG was accidentally discovered during a midurethral incision for a sling. The nodule containing the white CaHA material was excised, the sling surgery was cancelled, and the patient elected to have an alternate UBA injected [17]. Schrop et al. present a case of urinary retention caused by a partially obstructive CaHA FBG from an injection 50 months beforehand. During the excision procedure, the mass began to break apart and was described as whitish, chalky, gritty, and crystalline [18]. Elmhishi et al. describe a patient presenting with nausea, fatigue, and vomiting, with tests that showed hypotension, hypovolemia, urinary tract infection, and acute kidney injury. The bladder neck CaHA mass was obstructive to such a degree that it caused moderate bilateral hydronephrosis, and the Foley catheter inserted drained 800 mL urine immediately. The surgical treatment plan was not specified [19].

Additionally, we wish to draw attention to a post approval clinical trial of CaHA conducted between 2008 and 2015, which has since remained unpublished outside of FDA-mandated reporting requirements due to an agreement between the investigators and corporate sponsor [10]. The study is the largest ever of CaHA with 298 patients completing the full 36-month study duration, most of whom received one or two injections. The adverse event rate was alarmingly high, at 35.5% of patients for serious adverse events and 53.6% of patients for other (excluding serious) adverse events. The most common cases were urinary tract infection, urge incontinence, urinary retention, and micturition urgency. Stamey grade and quality of life scores both displayed a trend of sharp improvements from baseline to 6 months followed by an even plateau to the end of the study.

The MAUDE adverse events database contains two potentially relevant complication reports. The first patient received three sequential 1.0 mL CaHA injections. Sometime during the next calendar year, the patient was diagnosed with urethral prolapse via periurethral exam, which was assessed by the physician to be of mild severity and not CaHA-related. The urethral prolapse was untreated and resolved shortly. The patient was later diagnosed with caruncle via periurethral exam, which was assessed to be of mild severity and probably not CaHA-related. The caruncle was untreated and resolved shortly [20]. The second patient was unable to urinate without a catheter for 10 days after the CaHA injection, and experienced hematuria with loss of bulking agent particles into the urine due to a urethral tear [21]. Both patients were not formally included as the presence of an FBG requiring surgical intervention was inexplicit.

## Discussion

This review uniquely analyzes all known prior cases of CaHA-induced FBGs. To the best of the authors’ knowledge, no FBG adverse events have been reported in any clinical trials or retrospective studies of CaHA for SUI. The development of an obstructive FBG does not appear to be related to injection position, volume injected, or number of injections. While UBAs are typically injected around the proximal urethra, CaHA can migrate far away as seen by the even distribution of CaHA FBGs throughout the urethra. Although the rarity of this complication makes drawing conclusions between granuloma morphology and presenting symptoms difficult, the CaHA FBGs closer to the bladder neck were more likely to cause difficulty voiding or dyspareunia, whereas FBGs in the midurethra and distal urethra tended to cause renewed urinary incontinence. All three distal urethral FBGs were concomitant with urethral prolapse, possibly due to the weakening of the connection between the urethral mucosa and the underlying muscle near the meatus. The migratory ability of UBAs is supported by prior ultrasound literature, which showed that 41% of UBAs injected migrate and track away from the initial site and that 46% of patients have greater than 1 cm between the 3 o’clock and 9 o’clock implants [22]. CaHA particles manufactured to be slightly larger may be less likely to migrate and could help preserve the round implant shape that is necessary for proper coaptation. The ideal UBA should have a high degree of biocompatibility, producing enough of an inflammatory and fibrotic response to lock the microspheres in place but not to such an extent that an obstructive mass is produced [4].

While most of the cases involved granuloma discovery within 7 months of injection, the 20-month and 50-month cases should serve as a clinical reminder that this complication can manifest many years later. Due to large variability in FBG development times, pertinent observation should be conducted after UBA injection to monitor for any major complications that require surgery. The post-injection FBG risk may be higher in patients who recently had extensive urogenital surgery, as evidenced by our patient and multiple others [14–16]. Patients should be informed of all possible adverse events and counseled that further SUI procedures may be necessary, especially if an FBG develops and is resected. Additionally, the complications presented in this report highlight the importance of proper injection technique. Injections that are too superficial, deep, distal, proximal, or forceful may predispose the particles to migration, and healthy periurethral tissue is important to provide proper support for the implant [16]. Other clinical guidelines to consider if a UBA-related FBG is suspected are to utilize a Coude catheter and flexible cystoscope to better navigate the obstructed urethra. A longitudinal incision of the FBG to explant the beads prior to resection may be helpful as it preserves the urethral mucosa, protects the underlying musculature, and makes the suturing process easier.

Prior literature utilizes an imprecise nomenclature to describe suburethral masses which arise after UBA injection, consisting of FBG, pseudocyst, pseudoabscess, complicated abscess, and periurethral abscess. De Vries et al. found that at least one of these rare mass-related complications has occurred for every UBA except Urolastic, which has sparse clinical data [6]. Greater clarity in the characterization of these terms is necessary to better inform UBA treatment guidelines, as case reports or clinical studies often fail to provide gross or histological evidence to justify their classification. One of the harsher terms listed above are generally utilized when a large mass causes severe LUTS, whereas smaller, asymptomatic masses of identical biological composition are often labelled more mildly as nodules or saccules [11,17].

The foreign body reaction is formally defined as an aggregation of macrophages and multinucleated giant cells at the interface between a biomaterial and native tissue, and it can be accompanied by an inflammatory response [8]. Particulate UBAs always rely on the foreign body reaction to achieve urethral coaptation even for successful injections. Histological results in rabbit laryngeal tissue showed severe giant cell aggregation (over 50% of slide area), light levels of lymphocytic inflammatory infiltrate, mild fibrosis, and mild angiogenesis at both 3 and 12 weeks after CaHA injection. Mucosal inflammation was mildly prevalent at 3 weeks but normalized at 12 weeks [23]. Similar results were obtained when CaHA was used as a facial filler. Macrophages and giant cells persisted even at 24 weeks, and there was an initial transient inflammatory response caused mainly by histiocytes [24]. These findings of a long-term foreign body reaction even in the ordinary bulking process suggest that the term FBG should represent a gradient more than a binary classification. It is possible that the prevalence CaHA-induced FBGs is much higher than suggested by this review, as asymptomatic granulomas may never be discovered or labeled as such. Physicians should even consider leaving accidentally discovered, asymptomatic FBGs untouched as they can contribute to urethral coaptation if correctly positioned [17].

Despite the moderate improvements in quality of life scores and the promising lack of symptom rebound over time, the very high serious and nonserious adverse event rates reported in the unpublished post approval clinical trial demonstrate a need for more widespread clinician and patient discussion of these results while considering CaHA injection. Although claims regarding the relative efficacy and safety of various UBAs are beyond the scope of this review, clinical trials of other UBAs such as Bulkamid® and Contigen® have reported far lower rates in both adverse event categories with similar FDA definitions [25].

The main limitation of this literature review is the small sample size of known CaHA-related foreign body granuloma cases, which makes drawing conclusions about the condition’s etiology and subtle properties challenging. Greater reporting of UBA complications both in the scientific literature and in databases such as MAUDE is essential for sound physician response to future occurrences as well as proper choice of UBA type depending on patient history.

Patients and radiologists should be made aware that UBAs can often elusively manifest in future radiographic imaging, as improper diagnoses can lead to unnecessary treatments [26]. Gaines et al. report that only 39% of clinical images associated with urethral injection therapy were correctly interpreted by radiology, with the most popular incorrect diagnoses being bladder calculus, urethral calcifications, diverticulum, hypodense area, unknown nodular density, and mass suspected to be cancerous [27]. UBAs can migrate not only within the urethra but also to the cervicovaginal junction and other distant lower genitourinary regions [28]. Healthcare teams should also know that radiographic findings in patients with prior urethral UBA therapy may be misleading, as even in our present case the CT scan of the abdomen was suggestive of calcifications or stones. MRI signal intensity parameters and lesion characterization can be reliably used to differentiate UBAs from other lower urinary tract malformations [29]. The cases included in this review tended to describe the FBGs as suburethral crystalline or calcified ball-shaped objects with a whitish hue. These granulomas can cause severe BOO, urinary retention, and even bilateral hydronephrosis if treated too late, so excision is crucial if the urethra is obstructed.

Severe, persistent LUTS such as difficulty voiding, recurrent urinary incontinence, or dyspareunia after CaHA injection may indicate an FBG, which should be surgically resected. The rare development of CaHA-induced FBGs suggests the need for more biocompatible UBAs as well as greater patient and radiologist awareness of the potential adverse events and complications of UBA usage.

## Abbreviations

SUI Stress urinary incontinence; ISD Intrinsic sphincter deficiency; MUS Midurethral Sling; UBA Urethral bulking agent; CaHA Calcium hydroxylapatite; LUTS Lower urinary tract symptoms; FBG Foreign body granuloma; PVR Post-void residual; UUI Urge urinary incontinence; BOO Bladder outlet obstruction; TUR Transurethral resection.
